# Opportunities and challenges for research on food and nutrition security and agriculture in Asia Opportunities for future research and innovation on food and nutrition security and agriculture – a global perspective

**DOI:** 10.5713/ajas.18.0002B

**Published:** 2018-10-23

**Authors:** Jong K. Ha

**Affiliations:** 1 Asian-Australasian Journal of Animal Sciences, Seoul 08776, Korea; 2 Department of Agricultural Biotechnology, College of Agriculture and Life Science, Seoul National University, Seoul 08826, Korea

It is well recognized that national academies of science have a long tradition of engaging widely to strengthen the evidence base to underpin the delivery of enhanced food and nutrition security at regional and national levels. The Association of Academies and Societies of Sciences in Asia (AASSA) has recently produced a report for audiences of the Asia-Pacific regions as a contribution to a project worldwide initiated by the InterAcademy Partnership (IAP), the global network of science academies. The IAP work brings together regional perspectives in parallel from Africa, Asia, the Americas and Europe on the opportunities for the science-policy interface, identifying how research can contribute to resolving challenges for agriculture, food systems and nutrition, of which livestock production constitutes a very important component.

This **AASSA report on Food and Nutrition Security and Agriculture (FNSA)** focusses on FNS in the Asia/Oceania region, but given the size and importance of the region and the considerable inter-connectedness of matters influencing food production, food consumption, nutrition and human well-being, many of the observations and recommendations made are global in nature. The target outcome of its deliberations is future FNS, that is, access for everybody to a diverse healthy diet that is underpinned by a production/distribution/consumption system that is sustainable, environmentally, socially and culturally. It is recognized that there are many facets to FNS, and a systems approach to analysis is recommended, where the complex interactions between scientific and technical (physico-chemical, biological, environmental), economic, political, social and cultural dimensions are considered together. Having said this, in preparing this report the working group, and reflecting its expertise and mandate, has chosen to focus on the crucial role to be played by S&T (both R&D and education) in securing future FNS. Increasing pressures from population growth, urbanization, land availability, resource and water availability, pollution, global climate change, biodiversity loss conspire to make FNS a formidable near-term challenge.

The report identified key S &T areas having universal and prioritized application across the region as followings: (1) Genomic-based approaches to plant and animal breeding. (2) Big-data capture and analysis, precision agriculture, robotics. (3) Food technology innovations in harvest, processing and storage to reduce food wastage. (4) Sustainable farming practices for land and water use, that address wider issues such as biodiversity and climate. (5) Aquaculture production and integrated farm production systems.

**The Global Report from IAP**, analysis and synthesis of four regional reports from Asia, Africa, America and Europe, addresses recommendations for international scientific priorities as follow:

1. Developing sustainable food and nutrition systems, taking a systems perspective to deliver health and well-being, linked to transformation towards the circular economy and bio-economy.
2. Emphasising transformation to a healthy diet and good nutrition.
3. Understanding food production and utilisation issues, covering considerations of efficiency, sustainability, climate risks and diversity of resources.
4. Capitalising on opportunities coming within range in the biosciences and other rapidly advancing sciences
5. Addressing the food-energy-nutrients-water-health nexus, recognising that boundaries are blurred.
6. Promoting activity at the science-policy interfaces and reconciling policy disconnects. involving them in strategic decisions about planning research.
7. Consolidating and coordinating international science advisory mechanisms.

It is a must to readers who are looking for an up-to-date and comprehensive study with scientific and technological perspective on challenges and opportunities of food and nutrition security and agriculture both in Asian and global level. These reports can be enjoyed through websites: http://www.interacademies.org/37646/Food-and-Nutrition-Security-and-Agriculture.

Finally, the reviewer would like to congratulate IAP and AASSA for producing well-integrated and resourceful reports with STI perspective on FNSA of both Asia and Global status.

